# Geriatric syndromes in elderly hospitalized patients in China: a cross-sectional study

**DOI:** 10.3389/fmed.2026.1734756

**Published:** 2026-02-11

**Authors:** Xiaoyi Huang, Wei Cai, Hua Ren, Jinyi Sun, Xiaofen Pang

**Affiliations:** 1 Department of Geriatrics, Ruijin Hospital Lu Wan Branch, Shanghai Jiaotong University School of Medicine, Shanghai, China; 2 Occupational Disease Diagnosis and Treatment Center, Shanghai Institute of Occupational Disease for Chemical Industry, Shanghai, China

**Keywords:** China, geriatrics, polypharmacy, risk of falling, sleep disorders

## Abstract

**Introduction:**

Geriatric syndromes are nonspecific symptoms and signs that occur with aging. This study aims to investigate the prevalence of geriatric syndromes among elderly hospitalized patients in China.

**Method:**

This cross-sectional study conducted a Comprehensive Geriatric Assessment (CGA) on elderly patients hospitalized in Ruijin Hospital Luwan Branch, between January 2023 and July 2024. The CGA included evaluations of pain, sleep, constipation, fall risk, urinary incontinence, polypharmacy, nutritional risk, and dementia.

**Result:**

A total of 150 patients were included in this study. The five most prevalent geriatric syndromes were sleep disorders (44.67%), polypharmacy (40.67%), fall risk (24.67%), urinary incontinence (21.33%), and malnutrition (20.67%). The fall risk was significantly higher in women compared to men (37.50% vs. 12.82%, *p* < 0.001). Additionally, widowed individuals exhibited higher rates of fall risk (57.70% vs. 9.62%, *p* < 0.001) and malnutrition (47.83% vs. 8.65%, *p* < 0.001) compared to married individuals and other groups, with statistically significant differences.

**Conclusion:**

This study suggested that the incidence of sleep disorders, polypharmacy, and fall risk was relatively high among elderly hospitalized patients. Gender and marital status may have influenced the occurrence of these syndromes. Future care strategies could be tailored to specific populations.

## Introduction

Geriatric syndromes encompass a broad range of non-specific symptoms and signs that emerge as older adults experience functional decline across multiple organ systems with advancing age, without fitting into specific disease categories (1). Following the broad, operational definition adopted by the Chinese Geriatric Society (42) and Inouye et al. (2), we use the term “geriatric syndromes” to encompass pain, constipation, and sleep disturbances when they occur in interaction with age-related vulnerability. These syndromes commonly include gait abnormalities, chronic pain, urinary and fecal incontinence, Parkinson’s disease, delirium, falls, frailty, dizziness, syncope, and malnutrition (2, 3). They are not attributed to a single cause but result from progressive dysfunction across various systems, influenced by physical diseases, psychological conditions, social factors, and environmental elements (4). Geriatric syndromes significantly impact activities of daily living (ADLs) and increase vulnerability to morbidity and mortality in older adults (5). They also affect quality of life and psychological well-being, leading to progressive disability, greater caregiving needs, and markedly reduced life expectancy. The presence of geriatric syndromes can serve as important prognostic indicators for morbidity and survival in older adults (6). With the growing aging population in China, comprehensive geriatric assessment (CGA) is increasingly recognized and implemented. This assessment utilizes a multidisciplinary approach to evaluate various aspects of an older adult’s health, including physical function, nutritional status, cognition, activities of daily living, fall risk, sleep, mental health, polypharmacy, frailty, pressure ulcers, and home environment (7–9). The information gathered through these evaluations enables the formulation of tailored intervention plans and integrated care strategies that address immediate and long-term care needs (10). These treatment plans aim to protect the health and functional status of older adults, thereby enhancing their quality of life.

A recent study of 779 older adults in the United States found that 82% had at least one geriatric syndrome (11). In a cohort of 1,705 Australians aged over 70, fewer than 10% of those aged 70–79 exhibited poor mobility, falls, urinary incontinence, frailty, or dementia, whereas this proportion rose to over 10% among those aged 84 and older (12). In 2020, the population of individuals aged 60 and above in China was approximately 264 million (13), and a study of 706 older Chinese adults revealed that 90.5% had at least one geriatric syndrome (14).

Many studies have investigated the prevalence of individual geriatric syndromes (15–17), but up-to-date, multicentre data from mainland China using uniform CGA tools are still scarce, and regional estimates vary widely due to heterogeneous definitions and assessment methods. Understanding the prevalence of geriatric syndromes is crucial for developing effective care strategies for specific subgroups. Therefore, this study aimed to investigate the prevalence of geriatric syndromes among elderly hospitalized patients in China, providing a reference for subsequent integrated management.

## Methods

### Study design and patients

This cross-sectional study included older adult patients admitted to the Geriatrics Department of the Ruijin Hospital Luwan Branch, affiliated with the Shanghai Jiao Tong University School of Medicine, between January 2023 and July 2024, All patients aged ≥ 60 y admitted to the Department of Geriatrics during the study period were screened consecutively for eligibility, for diseases other than geriatric syndromes. This study was approved by Ruijin Hospital Luwan Branch Ethics Committee Shanghai JiaoTong University School of Medicine (LWEC2020025), and all participants provided written informed consent. After an amendment approved by the Ethics Committee on 03-Mar-2023 (LWEC2020025-A1) the analytical cohort was restricted to patients aged ≥ 80 y because of ward re-organisation.

The inclusion criteria were (1) patients aged 60 years or older and (2) those who provided informed consent. The exclusion criteria were (1) participation in other clinical trials, (2) abnormal mental status, (3) severe comorbidities (such as end-stage disease or critical illness), (4) severe dementia or (5) complete disability.

We acknowledge that excluding patients with severe dementia or altered mental status may underestimate the true prevalence of cognitive impairment and other syndromes. Consequently, our figures should be interpreted as a lower-bound estimate for the population able to participate in structured interviews.

### Data collection

All geriatric assessments were completed within 24 h of admission (median 18 h, IQR 12–23) to minimise hospital-related confounding.

The questionnaires were distributed, collected, inspected, and evaluated by healthcare professionals who had received standardized training. The questionnaire included sections on general information and the assessment of geriatric syndromes. The general information section assessed variables such as gender, age, marital status, height, weight, smoking, alcohol consumption, education level, occupation, and hobbies. The geriatric syndromes assessed included pain, evaluated using the Numeric Rating Scale (NRS) (18); sleep quality, assessed using the Pittsburgh Sleep Quality Index (PSQI) (19); constipation, assessed using the Chronic Constipation Assessment (20); fall risk, assessed using the Morse Fall Scale (21); urinary incontinence (22); polypharmacy, assessed using the STOPP/START criteria (23); nutritional risk, assessed using the Nutritional Risk Screening (NRS-2002) (24); and cognitive function, assessed using the Mini-Mental State Examination (MMSE) (25). If an individual exhibited any one of the above symptoms, they were diagnosed with geriatric syndrome. The specific assessment criteria are as follows:

The NRS for pain was used to assess pain intensity, with scores assigned as follows: 5 points for extreme pain, 4 points for very severe pain, 3 points for severe pain, 2 points for moderate pain, 1 point for mild pain, and 0 points for no pain.

The PSQI was used to assess sleep quality over the past month, evaluating factors such as sleep duration, efficiency, difficulty falling asleep, nighttime awakenings, and daytime dysfunction (e.g., daytime fatigue). PSQI scores ranging from 0 to 5 indicate good sleep quality, while scores greater than 5 suggest the presence of sleep disorders.

The Chronic Constipation Assessment was used to evaluate bowel movement frequency, stool consistency, and defecation difficulty. Key factors include the frequency of weekly bowel movements, stool hardness (assessed using the Bristol Stool Form Scale), and the need for assistance in defecation (e.g., medication or enema).

The Morse Fall Scale was used to assess fall risk based on factors such as (1) history of falls (0 or 25 points), (2) multiple diagnoses (0 or 15 points), (3) use of assistance devices (0–30 points), (4) medication (20 points for IV or special medications), (5) gait/mobility (0–20 points), and (6) mental status (0 or 15 points). Total scores of 0–24 indicate no risk, 25–50 indicate low risk, and >50 indicate high risk.

Urinary incontinence was screened with the validated International Consultation on Incontinence Questionnaire-Short Form (ICIQ-SF); participants scoring ≥1 were classified as incontinent and the subtype (stress/urge/mixed) was extracted from ICIQ-SF item 3. The Chinese version (Cronbach’s *α* = 0.88, test–retest r = 0.91) was employed (22).

Polypharmacy was assessed using the STOPP/START criteria, focusing on duplicate medications, unnecessary drugs (which may lead to drug interactions or adverse effects), missing essential medications, and the appropriateness of dosage and duration.

Malnutrition was assessed using the NRS-2002, which evaluates nutritional status based on disease severity, weight loss, and age. The scoring criteria include (1) disease severity (1–3 points), (2) nutritional status (0–3 points based on weight loss or BMI), and (3) an additional point for patients aged 70 or above. A total score greater than 3 indicates a risk of malnutrition.

The Mini-Mental State Examination (MMSE) was used to evaluate cognitive function across domains such as orientation, memory, attention, calculation, recall, and language. MMSE scores of 24–30 indicate normal cognition, 20–23 indicate mild cognitive impairment, 10–19 indicate moderate cognitive impairment, and scores below 10 indicate severe cognitive impairment.

#### Sample size

The final sample size (*n* = 150) was determined by the number of eligible patients admitted during the 6-month recruitment window. No a-priori power calculation was performed because the study was exploratory and descriptive, consistent with STROBE recommendations for observational prevalence surveys.

### Statistical analysis

Data were processed and analyzed using SPSS 22.0 (IBM, Armonk, NY, United States). Categorical data were expressed as *n* (%) and analyzed using the chi-squared (χ2) test. For *n* > 30 and <5, the Fisher’s exact test was used. Statistical significance was set at *p* < 0.05.

Multivariable logistic regression was performed for the two primary outcomes—fall risk and malnutrition—with simultaneous entry of age (continuous), sex (female vs. male), education level (≤ primary vs. ≥ primary), marital status (widowed vs. married/others), number of medications (≥ 6 vs. ≤ 6), and Charlson Comorbidity Index (continuous). Adjusted odds ratios (aOR) with 95% confidence intervals (CI) are reported in Table 1. A two-tailed *p* ≤ 0.05 was considered statistically significant.

**Table 1:** Multivariable logistic regression analysis of factors associated with fall risk and malnutrition.

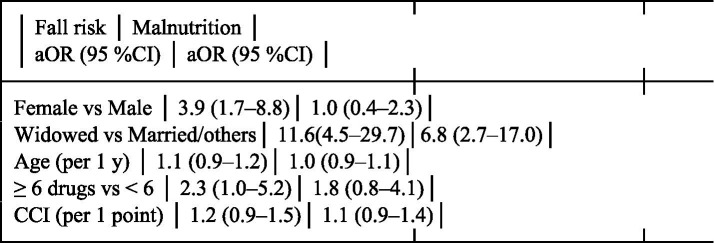

This manuscript was prepared in accordance with the STROBE guideline for cross-sectional studies. The completed STROBE checklist is provided as Supplementary Material 1.

## Results

**Figure 1:**
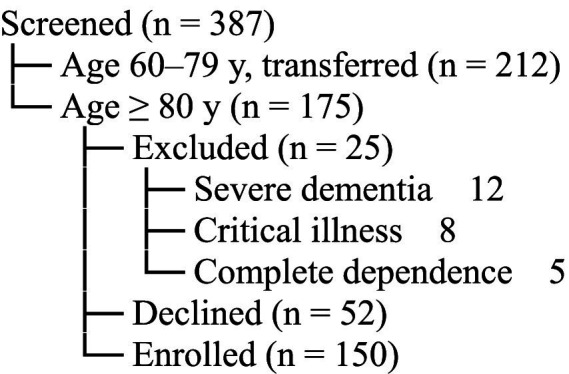
Trial profile of screened, excluded, and enrolled participants aged ≥ 80 y.

Figure 1 shows the flow of participants. During the 6-month recruitment period (January 2023–July 2024), 387 consecutive patients aged ≥ 60 y admitted to the Department of Geriatrics were screened for eligibility; 212 were aged 60–79 y and transferred to the general-medicine ward after an amendment approved on March 03, 2023, leaving 175 patients aged ≥ 80 y. Of these, 25 did not meet inclusion criteria (severe dementia *n* = 12, critical illness *n* = 8, complete disability *n* = 5), and 52 declined to participate. Consequently, 150 patients were enrolled and completed the assessment.

Figure 1 shows the trial profile. During the 19-month recruitment period (January 2023–July 2024), 387 consecutive patients aged ≥ 60 y admitted to the Department of Geriatrics were screened; 212 were aged 60–79 y and transferred to the general-medicine ward after the 03-Mar-2023 amendment, leaving 175 patients aged ≥ 80 y. Of these, 25 did not meet inclusion criteria (severe dementia *n* = 12, critical illness *n* = 8, complete functional dependence *n* = 5) and 52 declined to participate. Consequently, 150 patients were enrolled and completed the assessment (Figure 1). The most prevalent geriatric syndrome was sleep disorders (44.67%), followed by polypharmacy (40.67%), fall risk (24.67%), urinary incontinence (21.33%), malnutrition (20.67%), pain (15.33%), chronic constipation (14.67%), and dementia (7.33%) (Table 2). These 11 participants had mild-to-moderate dementia (CDR ≤ 2) and retained decisional capacity after bedside assessment (MMSE ≥ 17 plus UBACC ≥ 14.5); written consent was obtained directly (*n* = 7) or from a legal proxy (*n* = 4).

**Table 2:** The clinical characteristics of the study population.

Clinical data	Amount (*N* = 150)	Percentage (%)
Sex		
Male	78	52
Female	72	48
Age (years)		
80–85	45	30
86–90	81	54
>90	24	16
Income (RMB)		
<5,000	74	49.3
5,000–6,000	52	34.7
>6,000	24	16
Marital status		
Widowed	46	30.7
Married and others	104	69.4
Geriatric syndromes		
Sleep disorders	67	44.67
Polypharmacy	61	40.67
Chronic constipation	22	14.67
Fall risk	37	24.67
Urinary incontinence	32	21.33
Pain	23	15.33
Malnutrition	31	20.67
Dementia	11	7.33

The rate of fall risk was higher in females than in males (37.50% vs. 12.82%, *p* < 0.001) and in widowed individuals than in marrier and others (57.70% vs. 9.62%, *p* < 0.001). The rate of malnutrition was higher among widows than in married or other individuals (47.83% vs. 8.65%, *p* < 0.001). Age and income were not associated with any of the geriatric syndromes assessed in this study (all *p* > 0.05) (Table 3; Table 4; Supplementary Tables 1–6).

**Table 3:** Comparison of characteristics in patients with Fall risk.

Variable	*n* (%)	Z/χ^2^	*P*
Sex		12.272	<0.001
Male	10 (12.82)		
Female	27 (37.50)		
Age (years)		1.745	0.080
80–85	9 (20.00)		
86–90	17 (20.99)		
>90	11 (45.83)		
Income (RMB)		0.192	0.847
<5,000	17 (22.97)		
5,000–6,000	15 (28.85)		
>6,000	5 (20.83)		
Marital status		41.344	<0.001
Widowed	27 (57.70)		
Married and others	10 (9.62)		

**Table 4:** Comparison of characteristics in patients with malnutrition.

Variable	*n* (%)	Z/χ^2^	*P*
Sex		0.002	0.961
Male	16 (20.51)		
Female	15 (20.83)		
Age (years)		0.880	0.379
80–85	8 (17.77)		
86–90	16 (19.75)		
>90	7 (29.17)		
Income (RMB)		0.563	0.573
<5,000	14 (18.92)		
5,000–6,000	11 (21.15)		
>6,000	6 (25.00)		
Marital status		29.849	<0.001
Widowed	22 (47.83)		
Married and others	9 (8.65)		

## Discussion

The results of this study revealed a relatively high prevalence of sleep disorders, polypharmacy, and fall risk among elderly hospitalized patients. Furthermore, gender and marital status appear to influence the prevalence of these geriatric syndromes.

The increased prevalence of geriatric syndromes in the elderly is closely linked to the decline in organ function that accompanies aging, as well as the high prevalence of multiple chronic conditions (26). The findings of this study align with previous research, which have shown that geriatric syndromes are common among older adults. For instance, studies have reported that 90.5% of elderly individuals experience at least one geriatric syndrome, and 72.8% suffer from multiple syndromes (27, 28). In this study, the primary geriatric syndromes detected in elderly hospitalized patients were sleep disorders, polypharmacy, and fall risk. The main contributing factors to these results may include the prevalence of chronic diseases, multimorbidity, and the decline in function and cognition commonly seen in older adults. Sleep disorders are particularly prevalent in the elderly population. Age-related changes in circadian rhythms lead to a reduction in melatonin secretion and lower growth hormone levels, which disrupt sleep patterns. Most elderly individuals experience an advanced biological clock, contributing to sleep disturbances and regulatory issues (29). Additionally, the effects of chronic diseases or medication side effects cause gradual organ degeneration, making older adults more susceptible to conditions such as cardiovascular diseases and migraines, both of which can exacerbate sleep disorders. Research has shown that the prevalence of sleep disorders in elderly individuals is significantly higher compared to other age groups, with rates reaching up to 60% (30, 31).

At the time of PSQI administration, median length of stay (LOS) was 1 day (IQR 1–2). To examine whether acute hospital environment biased the results, we repeated the analysis after excluding 14 patients whose LOS ≥ 3 days; the prevalence of poor sleep remained virtually identical (46% vs. 47%, *p* = 0.91), suggesting that acute hospital-related factors had minimal impact on the PSQI findings. Polypharmacy is also highly prevalent among the elderly, often due to the presence of multiple chronic conditions. Recent studies have found that the comorbidity rate among elderly hospitalized patients in China ranges from 40 to 80%, with some regions reporting rates exceeding 90% (32). Multicentre surveys from Beijing (87%), Shanghai (92%), and Guangzhou (76%) have reported that the comorbidity rate among elderly in-patients in China ranges from 40% to over 90% (33–35). On average, elderly individuals in China suffer from six chronic conditions, which predisposes them to polypharmacy. Approximately 50% of elderly patients take three medications simultaneously, and 25% use four to six medications (32). The decline in physiological reserves in older adults increases their vulnerability to drug side effects, narrowing the safety margin between therapeutic and toxic doses. Several factors contribute to the increased fall risk in elderly individuals, including vision impairments, osteoporosis, decreased muscle strength, impaired balance, and the side effects of medications. These factors compromise physical stability and significantly increase the likelihood of falls (36, 37). Interestingly, gender and marital status were found to be statistically significant in influencing fall risk. Female patients, for example, may have a higher risk due to the loss of estrogen after menopause, leading to osteoporosis and muscle atrophy, which in turn increases fall risk. Among elderly individuals aged 65 to 69, women were found to have a higher incidence of falls compared to men, and a similar trend was observed in those aged 80 and above (38). Additionally, elderly individuals living alone are at greater risk due to a lack of communication and interaction with others, which can lead to undiagnosed physical decline, including vision and hearing impairments, as well as gait and balance problems (39). The absence of social engagement and support can delay recognition and treatment of these issues, further increasing fall risk (40). Moreover, elderly individuals living alone often face challenges with nutrition, as the lack of family and social support may result in poor dietary habits and insufficient nutrient intake (39). The decline in physiological function exacerbates the risk of malnutrition (41), which may help explain the correlation between malnutrition and marital status observed in this study.

After multivariable adjustment, female sex and widowhood remained independently associated with both fall risk and malnutrition, suggesting that these factors are important targets for tailored geriatric interventions.

Additional strengths include < 1% missing data across all instruments and inter-rater reliability *κ* ≥ 0.85 for every geriatric syndrome assessed.

This study has several limitations. First, the single-centre design limits the generalisability of our findings to other settings. Second, the cross-sectional nature precludes causal inference and temporal assessment of geriatric syndromes. Third, by excluding patients with severe dementia or complete functional impairment we likely under-represented the frailest segment of the hospitalised older population and may have underestimated the true prevalence of geriatric syndromes (potential selection bias). Fourth, the analytical cohort was restricted to patients aged ≥ 80 y after an ethics-approved amendment, further narrowing external validity to younger-old in-patients. Fifth, although we added multivariable models, residual confounding from unmeasured variables (e.g., social support, caregiver availability) cannot be ruled out. Sixth, the sample size was determined by practical recruitment rather than a-priori power calculation, which may reduce precision of estimates for rarer syndromes. Finally, longitudinal multicentre studies are needed to confirm our cross-sectional associations and to test whether targeted comprehensive geriatric assessment interventions can reduce syndrome burden.

In this single-centre cross-sectional study, three out of four hospitalised older adults aged ≥ 80 y presented with at least one geriatric syndrome. Female sex and widowhood were independently associated with higher prevalence. Multicentre longitudinal studies are needed to confirm these associations and to test whether targeted CGA interventions can reduce syndrome burden.

## Data Availability

The original contributions presented in the study are included in the article/Supplementary material, further inquiries can be directed to the corresponding author.
