# Supplementary material for: Geriatric syndromes in elderly hospitalized patients in China: a cross-sectional study
Source: Front Med (Lausanne). 2026 Feb 11;13:1734756. doi: 10.3389/fmed.2026.1734756 (PMC12932529; doi:10.3389/fmed.2026.1734756)
Supplement: Supplementary file 1 [file Table_1.DOCX]

**Table S1.** Comparison of characteristics in patients with Pain

| **Variable** | **n (%)** | **Z/χ^2^** | **P** |
| --- | --- | --- | --- |
| Sex, n (%) |  | 0.076 | 0.782 |
| Male | 34 (43.59) |  |  |
| Female | 33 (45.83) |  |  |
| Age (years) |  | -0.239 | 0.811 |
| 80-85 | 17 (37.78) |  |  |
| 86-90 | 35 (43.21) |  |  |
| >90 | 15 (62.50) |  |  |
| Income (RMB) |  | 0.186 | 0.852 |
| <5000 | 34 (45.94) |  |  |
| 5000-6000 | 20 (38.46) |  |  |
| >6000 | 13 (54.17) |  |  |
| Marital status |  | 2.516 | 0.113 |
| Widowed | 25 (54.35) |  |  |
| Married and others | 42 (40.38) |  |  |

**Table S2.** Comparison of characteristics in patients with Sleep disorders

| **Variable** | **n (%)** | **Z/χ^2^** | **P** |
| --- | --- | --- | --- |
| Sex |  | 0.057 | 0.811 |
| Male | 31 (39.74) |  |  |
| Female | 30 (41.67) |  |  |
| Age (years) |  | 1.612 | 0.107 |
| 80-85 | 16 (35.56) |  |  |
| 86-90 | 30 (37.04) |  |  |
| >90 | 15 (62.50) |  |  |
| Income (RMB) |  | -0.280 | 0.779 |
| <5000 | 32 (43.24) |  |  |
| 5000-6000 | 18 (34.62) |  |  |
| >6000 | 11 (45.83) |  |  |
| Marital status |  | 0.217 | 0.641 |
| Widowed | 20 (43.48) |  |  |
| Married and others | 41 (39.42) |  |  |

**Table S3.** Comparison of characteristics in patients with Chronic constipation

| **Variable** | **n (%)** | **Z/χ^2^** | **P** |
| --- | --- | --- | --- |
| Sex |  | 0.519 | 0.471 |
| Male | 13 (16.67) |  |  |
| Female | 9 (12.50) |  |  |
| Age (years) |  | 1.791 | 0.073 |
| 80-85 | 5 (11.11) |  |  |
| 86-90 | 9 (11.11) |  |  |
| >90 | 8 (33.33) |  |  |
| Income (RMB) |  | 1.130 | 0.259 |
| <5000 | 9 (12.16) |  |  |
| 5000-6000 | 8 (15.38) |  |  |
| >6000 | 6 (25.00) |  |  |
| Marital status |  | 0.140 | 0.709 |
| Widowed | 6 (13.04) |  |  |
| Married and others | 16 (15.38) |  |  |

**Table S4.** Comparison of characteristics in patients with Urinary incontinence

| **Variable** | **n (%)** | **Z/χ^2^** | **P** |
| --- | --- | --- | --- |
| Sex |  | 0.062 | 0.798 |
| Male | 16 (20.51) |  |  |
| Female | 16 (22.22) |  |  |
| Age (years) |  | 1.933 | 0.053 |
| 80-85 | 7 (15.56) |  |  |
| 86-90 | 15 (18.52) |  |  |
| >90 | 10 (41.67) |  |  |
| Income (RMB) |  | 0.032 | 0.974 |
| <5000 | 15 (20.27) |  |  |
| 5000-6000 | 13 (25.00) |  |  |
| >6000 | 4 (16.67) |  |  |
| Marital status |  | 0.007 | 0.936 |
| Widowed | 10 (21.74) |  |  |
| Married and others | 22 (21.15) |  |  |

**Table S5.** Comparison of characteristics in patients with Polypharmacy

| **Variable** | **n (%)** | **Z/χ^2^** | **P** |
| --- | --- | --- | --- |
| Sex |  | 0.223 | 0.637 |
| Male | 13 (16.67) |  |  |
| Female | 10 (13.89) |  |  |
| Age (years) |  | 1.539 | 0.124 |
| 80-85 | 5 (11.11) |  |  |
| 86-90 | 11 (13.58) |  |  |
| >90 | 7 (29.17) |  |  |
| Income (RMB) |  | 1.018 | 0.308 |
| <5000 | 10 (13.51) |  |  |
| 5000-6000 | 8 (15.38) |  |  |
| >6000 | 6 (25.00) |  |  |
| Marital status |  | 0.216 | 0.642 |
| Widowed | 8 (17.39) |  |  |
| Married and others | 15 (14.42) |  |  |

| **Variable** | **n (%)** | **Z/χ^2^** | **P** |
| --- | --- | --- | --- |
| Sex |  | 0.204 | 0.652 |
| Male | 5 (6.41) |  |  |
| Female | 6 (8.33) |  |  |
| Age (years) |  | 0.684 | 0.494 |
| 80-85 | 2 (4.44) |  |  |
| 86-90 | 7 (8.64) |  |  |
| >90 | 2 (8.33) |  |  |
| Income (RMB) |  | -0.926 | 0.355 |
| <5000 | 7 (9.46) |  |  |
| 5000-6000 | 3 (5.77) |  |  |
| >6000 | 1 (4.17) |  |  |
| Marital status |  | 0.064 | 0.800 |
| Widowed | 3 (6.52) |  |  |
| Married and others | 8 (7.69) |  |  |

**Table S6.** Comparison of characteristics in patients with Dementia
